# Quantifying lung cancer heterogeneity using novel CT features: a cross-institute study

**DOI:** 10.1186/s13244-022-01204-9

**Published:** 2022-04-28

**Authors:** Zixing Wang, Cuihong Yang, Wei Han, Xin Sui, Fuling Zheng, Fang Xue, Xiaoli Xu, Peng Wu, Yali Chen, Wentao Gu, Wei Song, Jingmei Jiang

**Affiliations:** 1 Department of Epidemiology and Biostatistics, Institute of Basic Medical Sciences, Chinese Academy of Medical Sciences / School of Basic Medicine, Peking Union Medical College, Beijing, China; 2 Department of Radiology, Peking Union Medical College Hospital, Chinese Academy of Medical Sciences, Beijing, China; 3 Department of Radiology, Beijing Chao-Yang Hospital, Capital Medical University, Beijing, China

**Keywords:** Lung neoplasms, Tomography (X-ray computed), Prognosis, Precision medicine

## Abstract

**Background:**

Radiomics-based image metrics are not used in the clinic despite the rapidly growing literature. We selected eight promising radiomic features and validated their value in decoding lung cancer heterogeneity.

**Methods:**

CT images of 236 lung cancer patients were obtained from three different institutes, whereupon radiomic features were extracted according to a standardized procedure. The predictive value for patient long-term prognosis and association with routinely used semantic, genetic (e.g., epidermal growth factor receptor (EGFR)), and histopathological cancer profiles were validated. Feature measurement reproducibility was assessed.

**Results:**

All eight selected features were robust across repeat scans (intraclass coefficient range: 0.81–0.99), and were associated with at least one of the cancer profiles: prognostic, semantic, genetic, and histopathological. For instance, “kurtosis” had a high predictive value of early death (AUC at first year: 0.70–0.75 in two independent cohorts), negative association with histopathological grade (Spearman’s *r*: − 0.30), and altered expression levels regarding EGFR mutation and semantic characteristics (solid intensity, spiculated shape, juxtapleural location, and pleura tag; all *p* < 0.05). Combined as a radiomic score, the features had a higher area under curve for predicting 5-year survival (train: 0.855, test: 0.780, external validation: 0.760) than routine characteristics (0.733, 0.622, 0.613, respectively), and a better capability in patient death risk stratification (hazard ratio: 5.828, 95% confidence interval: 2.915–11.561) than histopathological staging and grading.

**Conclusions:**

We highlighted the clinical value of radiomic features. Following confirmation, these features may change the way in which we approach CT imaging and improve the individualized care of lung cancer patients.

## Key points

- Radiomics-based image features are not used in the clinic despite growing literature.
- We selected eight features that have been informative in lung cancer screening.
- They show additional value in revealing semantic, genetic, histopathological and prognosis heterogeneity.
- These features are also robust to repeat imaging, segmentation operator and algorithm.
- The findings provide new insights into individualized care of lung cancer patients.

## Background

More than one in ten human cancers occurs in the lung [1]. The biological, spatial, and temporal heterogeneity of lung cancer makes its clinical management a critical challenge [2]. Computed tomography (CT) provides a means to observe the lung noninvasively and visualize lesions macroscopically, which can be performed repeatedly when necessary; thus, CT is core to the diagnosis and treatment workflow of lung cancer. The advantage of the knowledge inherent in CT images, however, is not being fully applied in the clinic, as only a few semantic characteristics or simplistic metrics (e.g., diameter) are routinely used [3, 4]. In the research field, efforts have been made in the translation of images into quantitative features that describe tumor shape and texture characteristics, as well as the association of these features with known clinical endpoints. This approach, termed radiomics, provides a unique opportunity for the generation of innovative methods for phenotypic profiling of lung cancer [5]. Several studies have demonstrated the potential use of radiomics-based metrics to deepen our knowledge on how lung cancers differ from benign lesions [6], and how cancer types differ from one another regarding development [7], prognosis [8], treatment response, and recurrence [9].

There are several important barriers that impede the use of radiomic features clinically: (1) the majority of radiomic research reports on isolated results regarding specific tasks, such as cancer diagnosis or prognosis; thus far, the value of radiomic features has rarely been demonstrated in independent datasets and for multiple tasks [10, 11]; (2) many radiomic features are computationally complex and difficult to interpret; lack of consensus on the definition [12] and interpretation of the associations between specific radiomic features with known cancer phenotypes make it challenging for physicians to uptake [3]; and (3) considering the variations in imaging acquisition and processing, the percentage of radiomic features that are deemed reproducible is low, about 6–43% in phantom studies and 10% in patient studies [13].

Because it has been ~ 10 years since radiomics gained its name, it is a suitable time to consider the controversies between a rapidly growing body of publications and the clinical application [14]. In a previous proof-of-concept study, in which we determined the optimal time for diagnostic testing in lung cancer screening, we found that several radiomic features were robust to image noise, associated with semantic characteristics, and predictive of lung cancer diagnosis [15]. In the present study, we validate the clinical value of these radiomic features based on their ability to predict long-term prognosis of lung cancer patients. We also provide additional support for their clinical uptake by showing the associations of these features with semantic, genetic, and histopathological heterogeneity in lung cancer, as well as the measurement reliability of these features.

## Methods

### Data sources

The proof-of-concept study was conducted between 2014 and 2019 at Peking Union Medical College Hospital and included 92 patients enrolled in a lung cancer screening program [15]. The CT images used in this study were from 236 lung cancer patients at three other institutes [16, 17]. Cohort A: a total of 146 early-stage non-small cell lung cancer (NSCLC) patients were recruited between years 2008 and 2012 at Stanford University School of Medicine and underwent CT and/or positron emission tomography (PET)/CT scan before surgical treatment (three patients had no CT images and were excluded). Cohort B: CT images of 61 lung adenocarcinoma patients before surgical treatment were acquired between years 2006 and 2009 at H. Lee Moffitt Cancer Center. Cohort C: CT images were collected in 2017 at Memorial Sloan-Kettering Cancer Center and were from 32 patients with NSCLC who underwent two CT scans of the chest within 15 min.

The images were retrieved from the Cancer Imaging Archive (https://www.cancerimagingarchive.net). To reflect the diverse clinical settings of each clinical center, there was no attempt to harmonize the image acquisition protocols (the clinical settings of each center are summarized in Appendix Table 3). The data usage policy for the collection of each image was followed. There was no need for ethical approval because all the patient information was de-identified.

### Image segmentation and feature extraction

The process of image segmentation and feature extraction has been previously standardized [15] and was performed accordingly in this study.

In brief, regions-of-interest (ROIs) were delineated following an interactive process: cross-sectional slices with the maximum area per pulmonary lesion were selected, and the threshold method (for juxtapleural lesions or part-solid and non-solid lesions) or manual initial contouring (for complex cases) was exercised, followed by automated refinement using Image Segmenter Toolbox, MATLAB R2018a until visual satisfaction. Two investigators contoured the ROIs on the initial scan images to assess the between-operator variation, and one of the investigators used two segmentation algorithms—region-based and edge-based active contour models—to assess the between-algorithm variation.

Radiomic features were then extracted from the ROIs using online and in-house adapted codes of MATLAB software (see [15] for specific definitions). We evaluated two sets of radiomic features: (1) eight selected features, including a shape feature (circularity; quantifying the degree of the ROI approximating a circle), three statistical features (variance, kurtosis, and energy; quantifying the dispersion, sharpness and magnitude of ROI brightness, respectively), three texture features (cluster-shade, maximum-probability, and long-run high gray-level emphasis mean [LongHEM]; quantifying the asymmetry, predominance of coexisting image pixel pairs, or the degree of bright coarse structural textures, respectively), and a wavelet feature (long-run emphasis mean on approximation signal; reflecting the structural texture in a probably finer resolution); these features had undergone a rigorous selection process in the proof-of-concept study using the following criteria: robustness to artificial image noise, predictive performance of cancer diagnosis, and non-redundancy (i.e., small effect of collinearity between features); see [15] for detailed feature selection workflow; (2) 11 features that revealed similar clinical utility potential but correlated to one of the selected features. We present the eight selected features in the results section, and the remaining information is available in Appendix.

For comparison purposes, diameter was calculated as a classical image metric, which was calculated by the average of the major and minor axis lengths, rounded to the nearest integer; semantic characteristics of the cancer images (including solid, lobular, specular, juxtapleural, and pleura tags) were interpreted by a researcher and a radiologist experienced at reading chest CT images.

### Analytic workflow and methods

Data on survival outcomes were available from Cohorts A and B, and we used these two cohorts for examining the prognostic value of the radiomic features. We randomly divided Cohort A (considering its relatively large sample size) using a 2:1 ratio for the training and testing groups; independent external validation was then performed using Cohort B. A machine-learning random survival forest (RSF) model was used to build a composite radiomic score that could more comprehensively evaluate the cancer characteristics than a single feature. The RSF is an extension of random forest (ensemble of tree models) to survival outcome [18] and was implemented with R package “randomForestSRC,” with hyper-parameters (ntree = 50, nodesize = 10, nodedepth = 4) determined by grid search. The predicted risk for each patient was rescaled to 0–100 by normalization to convert it to a score. Prognosis values of the radiomic features and score were measured by C-statistic (overall discrimination of time-to-survival outcome), as well as at several points of interest (e.g., to determine 2- or 5-year survival), using time-dependent area under the receiver operating curve (AUCt). Hazard ratio (HR) of death was computed between groups of patients with high and low scores.

To determine a clinical explanation of the novel features, in the proof-of-concept cohort, we associated the radiomic features with the semantic characteristics that are widely used in lung cancer image interpretation [15]; we further validated these associations in Cohorts A, B, and C. We also used information available in Cohort A on gene mutations (epidermal growth factor receptor [EGFR], Kirsten rat sarcoma viral oncogene homolog [KRAS], and anaplastic lymphoma kinase [ALK]) and histopathology (types and grades) to confirm the findings biologically. The associations of the radiomic features with these micro-level subtypes were examined using a differential expression analysis approach using the Wilcoxon test and Spearman’s correlation.

Lastly, for comprehensive assessment of the measurement reliability of the radiomic features, we examined feature reproducibility in terms of image acquisition (between-repeat scans) and segmentation (between operators and between algorithms) using data from Cohort C. Features with an intraclass coefficient (ICC) ≥ 0.8 were considered robust to the abovementioned variation. Bland–Altman plots were drawn for visual analysis.

The statistical tests were two-sided, with a significance level of 0.05. All the statistical analyses were performed with R version 3.5.2.

## Results

### Patient characteristics

Across the three patient cohorts (Table 1), the characteristics of lung cancer did not vary significantly regarding location (31.3–40.6% in the left lung [*p* = 0.2765]) or semantic characteristics (65.6–68.9% were a lobular shape, 24.5–37.5% were a spiculated shape, 37.7–59.4% had pleural invasion [elastic, visceral, or parietal], and 15.6–23.0% had pleura tags [all *p* > 0.050]), with the exception of image intensity, which varied between cohorts (50.8–81.3%, solid intensity [*p* = 0.0155]). Between patients in Cohorts A and B, there were no significant differences regarding cancer stage: 17.5% vs 29.3% staged IIIA or above, respectively; or regarding survival: median survival time was 40.0 vs 62.8 months, respectively (both *p* > 0.050).

**Table 1:** Characteristics of lung cancer patients

Characteristics	Cohort A (*N* = 143)	Cohort B (*N* = 61)	Cohort C (*N* = 32)	*p* value
Male, *n* (%)	108 (75.5)	31 (50.8)	16 (50.0)	0.0004
Mean age, years (range)	69.3 (43–87)	NA^†^	62.1 (29–82)	–
*Cancer location*				0.2765
Left upper lobe	38 (26.6)	20 (32.8)	4 (12.5)	
Left lower lobe	20 (14.0)	8 (13.1)	6 (18.8)	
Right upper lobe	51 (35.7)	21 (34.4)	9 (28.1)	
Right middle lobe	13 (9.1)	5 (8.2)	3 (9.4)	
Right lower lobe	21 (14.7)	7 (11.5)	10 (31.3)	
*Semantic characteristics*				
Solid	90 (62.9)	31 (50.8)	26 (81.3)	0.0155
Lobular	96 (67.1)	42 (68.9)	21 (65.6)	0.9470
Spiculated	35 (24.5)	17 (27.9)	12 (37.5)	0.3218
Juxtapleural	59 (41.3)	23 (37.7)	19 (59.4)	0.1121
Pleura tag	28 (19.6)	14 (23.0)	5 (15.6)	0.6936
Cancer stage				0.0617
0-IIB	118 (82.5)	41 (70.7)^‡^	NA	
IIIA–IVB	87 (17.5)	17 (29.3)	NA	
Median survival (IQR), month	62.8 (45.9, 72.5)	40.0 (31.0, 49.0)	NA	0.0636

NA, not available; IQR, inter-quartile range

^†^Age < 65 years: *n* = 20; ≥ 65 years: *n* = 41; specific data unavailable

^‡^Data unavailable for three patients

### Prognosis value

When used alone, the feature, “LongHEM,” showed a prognostic value that was equal to diameter. The overall C-statistic was 0.627 (LongHEM) vs 0.570 (diameter) in Cohort A, and 0.602 vs 0.605 in Cohort B; AUCt for predicting survival beyond 2 and 5 years was 0.617 (LongHEM) vs 0.612 (diameter) and 0.588 vs 0.597, respectively, in Cohort A, and 0.648 vs 0.618 and 0.670 vs 0.678, respectively, in Cohort B. A similar prognostic value was observed regarding features “kurtosis,” “energy,” and “maximum-probability” (Appendix Table 4).

Next, we developed a composite score using the eight selected features. The prognostic value of the composite score (Appendix Table 5) was greater than diameter, the five semantic characteristics, and a combination of these routine characteristics. For instance, when used for predicting survival beyond 5 years, the AUCt was 0.855 (train), 0.780 (test), and 0.760 (external validation) for the composite score, vs 0.733 (train), 0.622 (test), and 0.613 (external validation) for a combination of diameter and semantic characteristics.

**Fig. 1:**
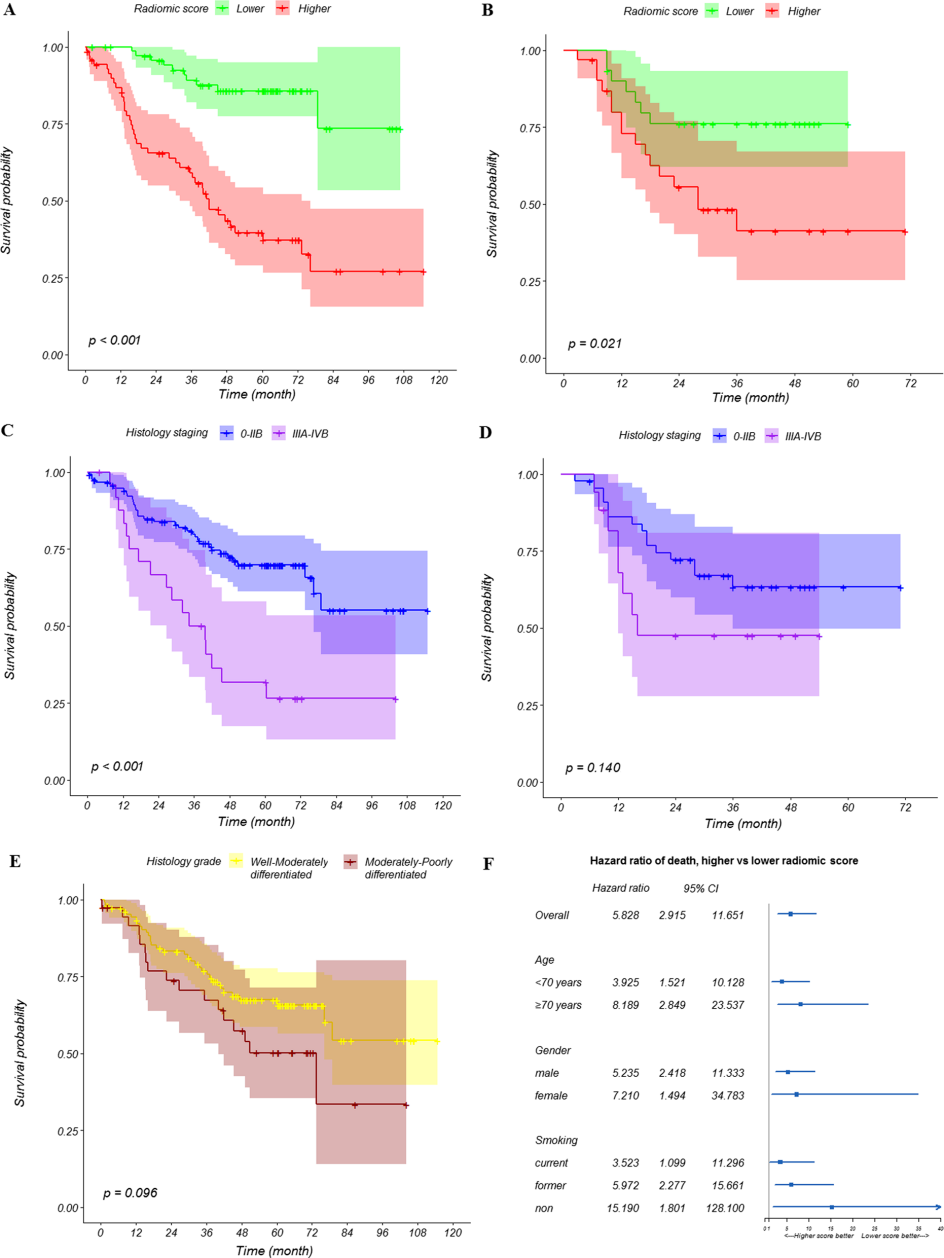
Survival of lung cancer patients. Stratified by composite radiomic score, (**A**) Cohort A, (**B**) Cohort B; histopathological staging, (**C**) Cohort A, (**D**) Cohort B; histopathological grading, (**E**) Cohort A (data not available for Cohort B); and by demographic subgroups for the examination of score value, (**F**) Cohort A

When patients in Cohorts A and B were stratified according to their median scores, we found significantly different prognosis between groups with low and high scores (*p* < 0.050 in both cohorts; Fig. 1A, B). Patients with a low radiomic score were associated with higher chances of survival from the beginning (1 year) of the follow-up process, and the between-group divergence in survival curves became more apparent after this time point. The performance of the radiomic score was better compared to histopathological staging (Fig. 1C, D) and grading (Fig. 1E; data only available for Cohort A). The HR of patients with a high vs low radiomic score was 5.828 (95% confidence interval [CI] 2.915–11.561) in Cohort A and 2.722 (95% CI 1.117–6.633) in Cohort B. The prognostic value was demonstrated across age, gender, and smoking category subgroups (Fig. 1F), and more pronounced among older (aged ≥ 70 years, HR 8.189), female (HR 7.210), and non-smoking patients (HR 15.190; all *p* < 0.050 against a null effect).

### Associations with semantic, genetic, and histopathological profiles

First, by associating the radiomic features with the semantic characteristics (Table 2), the following findings in the proof-of-concept cohort were independently confirmed in Cohort A: up-regulation of feature “maximum-probability” in cancers of solid intensity, of feature “variance” in cancers of spiculated shape, and of features “circularity” and “energy” in cancers attached to the pleura. We also found an association between “circularity” and juxtapleural location in Cohort B. Additionally, down-regulation of the feature “kurtosis” in juxtapleural cancers was demonstrated in Cohorts A and B, and up-regulation of feature “LongHEM” in juxtapleural cancers was observed in Cohorts A and C. The directions of the significant differentially expressed features were consistent across different cohorts, with the exception of “LongHEM” and spiculated shape.

**Table 2:** Association of selected radiomic features with semantic characteristics of lung cancer

Feature	Solid	Lobular	Spiculated	Juxtapleural	Pleura tag
Circularity			↓A^**^	↑P^*^;↑A^**^;↑B^*^	↓A^**^
Variance	↑A^**^		↑P^**^;↑A^*^	↓B^**^	↑A^*^
Kurtosis	↓A^**^		↓P^*^	↓A^**^;↓B^*^	↑A^**^
Energy	↑A^**^			↑P^**^;↑A^**^	↓A^**^
Cluster-shade		↓A^*^	↓P^*^	↑C^*^	↓B^*^
Maximum-probability	↑P^**^;↑A^**^	↓P^*^		↑A^**^	
LongHEM	↑A^**^		↑P^*^;↓C^*^	↑A^*^;↑C^**^	↑P^*^
A_Long-run emphasis	↓P^*^		↑A^**^		↓C^*^

↑ and ↓ denote up- and down-regulation of the feature in the presence of the semantic characteristics, respectively

A, B, C, P denote statistically significant (* *p* < 0.050; ** *p* < 0.010) association observed in cohorts A, B, C and the proof-of-concept cohort, respectively

LongHEM, long-run high gray-level emphasis mean

We then analyzed the relatively large sample size and the available information from Cohort A to evaluate radiomic expression patterns regarding different genetic and histopathological profiles.

**Fig. 2:**
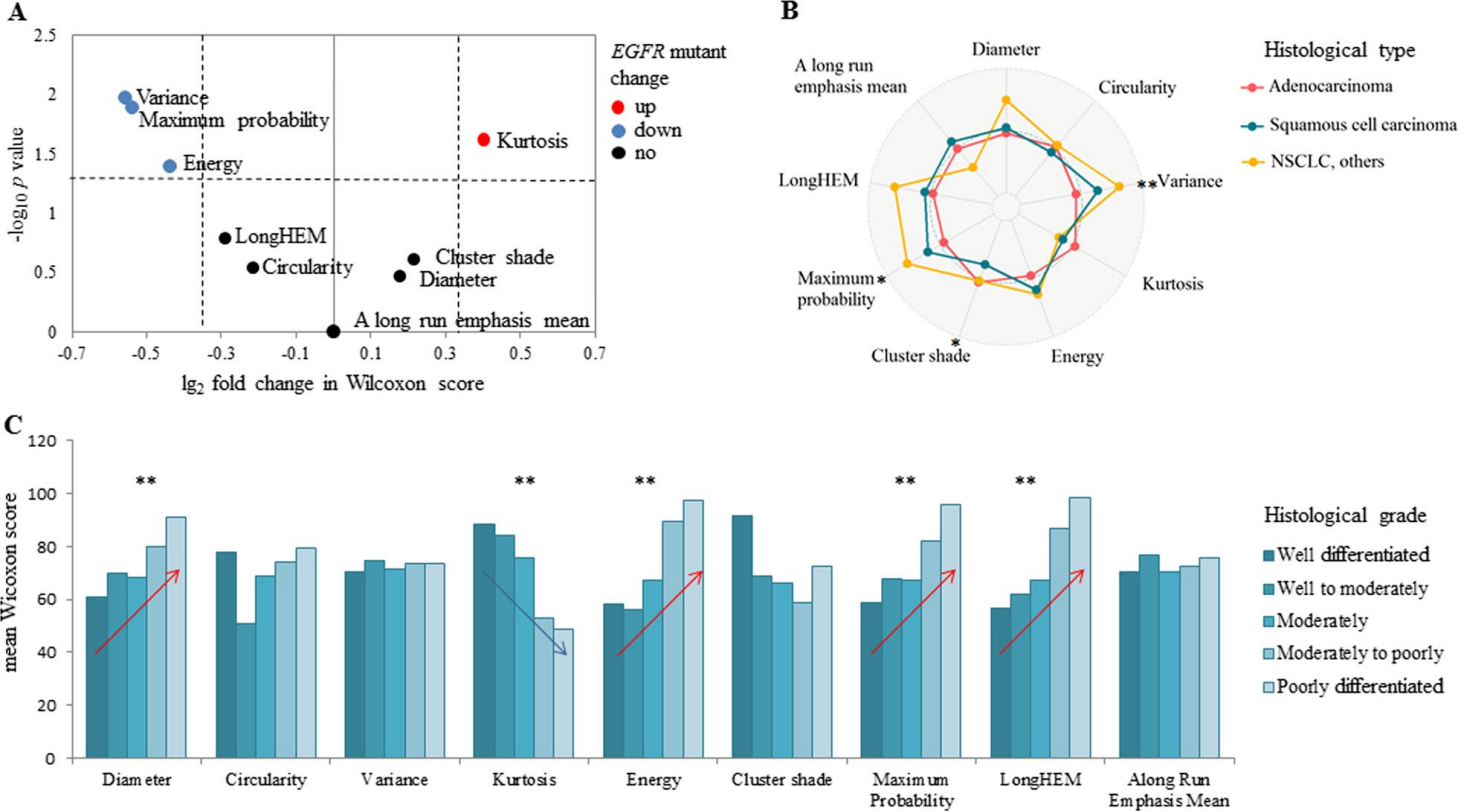
Association of image metrics with gene and histopathological phenotypes. **A** Volcano plot showing the significantly up-regulated (red) and down-regulated (blue) image metrics (*p* < 0.050) regarding EGRF mutation. **B** Radar plot of differentially expressed image metrics regarding histopathological type. **C** Dose–response plot of histopathological grade with image metrics. *p < 0.050; **p < 0.010. EGFR: epidermal growth factor receptor

EGFR-mutated (*n* = 23) relative to wild-type (*n* = 93) lung cancers showed an up-regulation of the feature “kurtosis” (median: 3.87 vs 2.48; *p* = 0.0238), and down-regulation of the features “maximum-probability” (0.53 vs 0.75; *p* = 0.0130) and “energy” (0.05 vs 0.09; *p* = 0.0401; Fig. 2A). Moreover, a down-regulation of feature “variance” was observed in ALK-translocated (*n* = 2) vs wild-type (*n* = 109) cancers (4.60 vs 8.64; *p* = 0.0282), but there was no differentially expressed features regarding KRAS-mutated (*n* = 27) vs wild-type (*n* = 88) cancers.

For cancers stratified by different histopathological groups (adenocarcinoma, squamous cell lung cancers, and other NSCLCs; Fig. 2B), the difference observed for the feature “variance” was highly significant (8.25 vs 8.94 vs 9.61; *p* = 0.0031). Other differentially expressed features regarding these histopathological groups included “cluster-shade” and “maximum-probability” (*p* < 0.050 for both of the features). Moreover, we found that four features significantly increased or decreased along with an increase in histopathological grade (Fig. 2C): “LongHEM” (Spearman’s coefficient of correlation: 0.331), “kurtosis” (− 0.329), “energy” (0.325), and “maximum-probability” (0.281; all *p* < 0.010).

Compared to the selected radiomic features, diameter had no discriminative power regarding cancers with EGFR or ALK mutations, nor for histopathological types (all *p* > 0.050). The correlation with histopathological grade (Spearman’s coefficient of correlation: 0.226; *p* < 0.010) was weaker than for the four aforementioned radiomic features (“LongHEM,” “kurtosis,” “energy,” and “maximum-probability”).

### Feature reproducibility

**Fig. 3:**
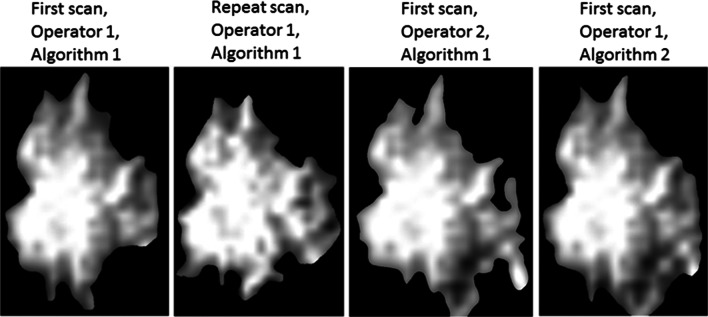
Segmented lung cancer images

**Fig. 4:**
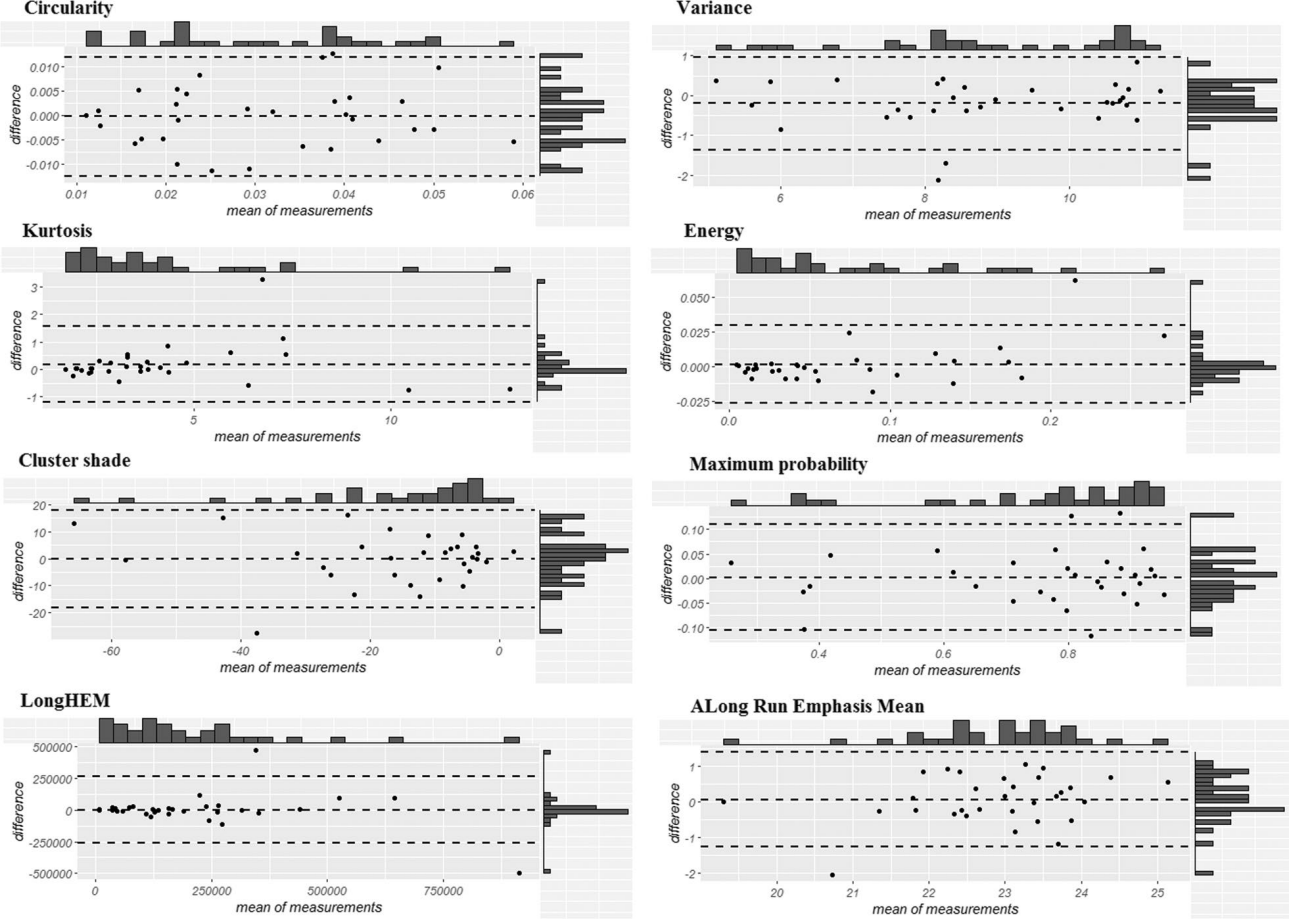
Reproducibility of eight radiomic features regarding measurements between scans

Despite the visual differences in the segmented ROIs between repeat scans and segmentation operators and algorithms (see Fig. 3 as an example), the eight radiomic features were generally not affected by these sources of variation (Fig. 4; Appendix Table 6). Specifically, ICC ranged from 0.81 (“LongHEM”) to 0.98 (“energy”) in repeat scans, and from 0.80 (“circularity”) to 0.99 (“kurtosis”) between segmentation operators and algorithms, with the exception of an ICC of 0.70, which was observed for between operators, and an ICC of 0.57, which was observed for between algorithms (“cluster-shade”).

## Discussion

Only a few quantitative or semantic features are routinely used in lung cancer image interpretation, and these features are considered insufficient because they are too simple or prone to inter-observer variability [19]. Currently, despite rapidly expanding literature on radiomics, there is no clinical use of radiomic features [20]. On the basis of our previous report [15] and the findings in the current study, we show that the eight selected radiomic features are predictive of both the diagnosis and prognosis of lung cancer, descriptive of semantic characteristics, and some are indicative of genetic and histopathological profiles. The selected features were largely robust to variation in image noise, repeat imaging, and visual differences in ROIs caused by segmentation operators and algorithms. When combined, the radiomic features showed a moderate prognosis value (AUCt for 5-year survival: 0.760 in external validation) and capability for risk stratification (HR 5.828 and 2.722 in two independent cohorts; the HRs among elderly, female, and non-smoking subgroups were 8.189, 7.210, and 15.190, respectively), and the stratification capability was even better than histopathological staging and grading. These results highlight the feasibility of using new metrics for lung cancer images in the clinical setting.

Because of the substantial heterogeneity in lung cancers, new methods for subtyping are needed. This issue has led to numerous efforts regarding cancer characterization at the tissue (imaging), cellular (microcopy), and molecular (genetic test) levels [5, 21]. Of these methods, histopathological methods are some of the oldest methods (specialty of histopathology techniques dates back to 1838 [22]), which laid a foundation for the diagnosis and treatment of cancer. Alterations to the cancer classification system are being pursued in the age of precision medicine [21], driven by improved methods that are able to reveal the molecular basis of the disease. The time for the introduction of medical imaging lied in between [9]; strengthened by a better computation power, description of the cancer morphology (and potentially its evolving biology) can go beyond human perceptions [23]. This may help to explain why the radiomic score outperformed the histopathological staging and grading in this study. Similar findings were observed in another study [24] that used more features for the construction of a radiomic signature than our study. Our finding on the interweaving associations between radiomic features and pathohistological and genetic profiles, as well as findings from several other publications (regarding prediction of lung cancer histopathology [25], EGFR and ALK mutations [26, 27], and prognosis among ALK-positive patients [28] using radiomic features), altogether confirm the presence of a connection between the macro-level imaging information and micro-level biology. As CT images are routinely collected, the analysis of radiomic features could be a relatively inexpensive and non-invasive means for cancer profiling.

Radiomic features are prone to variations in each step, from image acquisition to segmentation [29]. This is a key issue for the application of radiomics. For instance, many of the radiomic-semantic associations observed in our proof-of-concept study, validated in Cohort A or B, were not found in Cohort C. Despite the small sample size in Cohort C, we believe this discrepancy was due to the larger slice thickness (2.5–6.0 mm) in the samples in Cohort C compared with the other cohorts (0.6–3.0 mm). In a recent publication [13], slice thickness had the largest impact on feature reproducibility compared with other factors; slice thickness impacted visualization and interpretation of semantic characteristics (e.g., spiculated shape) according to our experience. We did not focus the present study on volumetric radiomic features or features requiring sophisticated quantification (such as those based on wavelet and other transformed images) because multiple previous publications [30, 31] and our proof-of-concept study [15] showed less reproducibility for these features than for shape, statistical, and a subset of texture features, even for peritumoral radiomics [31]. Instead, in the present study, following a rigorous selection process, we focused on several radiomic features. The feature reproducibility results confirmed that these features were reliable. Therefore, the analysis of these features may help to avoid spurious findings in subsequent analyses and in the clinical setting.

Among the selected radiomic features, “kurtosis” is a simple statistical feature describing the sharpness of the image intensity level distribution. Because of its simplicity, features of this category may be more robust than measuring shape and texture features (e.g., “circularity” and “cluster-shade” in this study), according to a systematic review [29]. For instance, “kurtosis” showed an ICC of > 0.96 between repeat scans and between segmentation operators and algorithms, which is relatively higher compared with other selected features. The clinical potential of the “kurtosis” feature as a new image metric for classification and progression of lung diseases has been repeatedly observed [32]. We further revealed several good properties of “kurtosis,” such as its high predictive value of early death (AUC at first year: 0.70–0.75 in two independent cohorts), negative linear association with histopathological grade (Spearman’s *r*: − 0.30), and significantly altered levels regarding EGFR mutations and regarding nearly all the semantic characteristics investigated. On the basis of these findings, we expect a wider application of this quantitative metric in the future given its ease-of-computation and interpretation.

Nevertheless, we could not expect too much from a single metric as a solution to a specific clinical task. For instance, in this study, the prognostic value of the radiomic features were, at best, equal to the use of diameter when used alone, and for most of the features, such values may vary over time. For some features, the temporal trend may also differ between cohorts (Appendix Table 4). Owing to their substantial heterogeneity, lung cancers of the same histopathological type can show varying imaging and survival characteristics; such variations have also been reported in cancers of the same genetic type [33]. Implications of these complex findings are far-reaching. First, these findings indicate that a lot more progress required in the pursuit for novel methods for cancer characterization. Regarding morphology, it was recently indicated that homology-based [34], peritumoral [35], or sequential radiomic features [36] may add to the value of standard radiomics, though further validations are needed. Second, to capture and differentiate tumoral heterogeneity, we need to take advantage of the effect of combined approaches. One example of this from the current study is that when the selected features (non-redundant measurements of cancer appearance) were combined as a score, their prognostic value was significantly enhanced. Therefore, mathematical formulas, statistical models, or machine learning algorithms deserve further investigation in this context [37]. Third, despite our enthusiasm for optimizing models’ precision for precise cancer care, we argue for an equal emphasis on basic techniques that are highly reproducible and allow adequate interpretation of the results.

There are several limitations in this study: (1) although digital images can be stored over long periods, the retrospective nature of this study limited our access to the demographic and genetic data of some patients; (2) CT images from more diverse scanning protocols should be considered for a better extrapolation of the results, and the impact of varying scanning protocols on feature reproducibility remain inadequately assessed; (3) the use of PET/CT images can improve the accuracy of cancer staging; therefore, it remains unknown whether the results on the prognosis value of the composite radiomic score against histopathological staging may be altered by adding such information; (4) we only examined a small number of selected radiomic features and applied them to a case-mix of lung cancer patients; this may have led to a lower prognostic accuracy compared with other reports [38, 39]; (5) the value of the selected radiomic features was only assessed at a certain time point (before surgery). It was indicated in our previous study that features such as “LongHEM” may be a good metric for monitoring purposes when measured repeatedly (i.e., delta radiomics); validation of this finding requires longitudinal data and further analysis.

## Conclusion

Following a previous work, we demonstrated in this study that several selected radiomic features are predictive of survival outcome and can differentiate between semantic, genetic, and histopathological heterogeneities in lung cancer. Moreover, the measurement of these features was reproducible in repeat scans and image segmentation. Following confirmation, the novel features described in this study may help to improve approaches of CT image analysis, and therefore, may improve individualized care of lung cancer patients.

## Figures and Tables

**Table 4:**
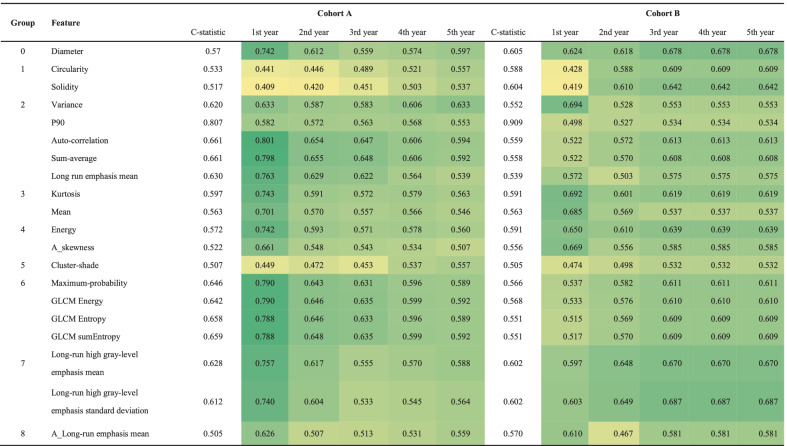
Overall and time-dependent performance of single image metrics

GLCM, Gray-level co-occurrence matrix

## Data Availability

The datasets supporting the conclusions of this article are available in the Cancer Imaging Archive repository: https://doi.org/10.7937/K9/TCIA.2017.7hs46erv. https://wiki.cancerimagingarchive.net/display/Public/NSCLC+Radiogenomics; https://doi.org/10.7937/K9/TCIA.2015.A6V7JIWX. https://wiki.cancerimagingarchive.net/display/Public/LungCT-Diagnosis; https://doi.org/10.7937/K9/TCIA.2015.U1X8A5NR. https://wiki.cancerimagingarchive.net/display/Public/RIDER+Lung+CT.

## Appendix

Tables 3, 4, 5 and 6.

**Table 3:** CT image acquisition protocols

	Cohort A	Cohort B	Cohort C
Data source	Memorial Sloan-Kettering Cancer Center	Stanford University School of Medicine	H. Lee Moffitt Cancer Center
Tube voltage	120 kVp	80–140 kVp	120–140 kVp
Tube current	298–441 mA	124–699 mA	14–384 mA†
Slice thickness	1.25 mm	0.6–3.0 mm	2.5–6.0 mm
Contrast enhancement	No	Yes/No	Yes/No

^†^With the exception of one using Ultravist contrast

**Table 5:** Overall and time-dependent performance of a composite radiomic score at predicting lung cancer prognosis

	Training set	Test set	External validation set
C-statistic	0.830	0.687	0.672
AUC (*t* = 1 y)	0.965 (NA)	0.811 (NA)	0.643 (0.459,0.800)
AUC (*t* = 2 y)	0.913 (0.832,0.978)	0.658 (0.496,0.789)	0.673 (0.515,0.819)
AUC (*t* = 3 y)	0.851 (0.781,0.937)	0.642 (0.473,0.781)	0.760 (0.628,0.911)
AUC (*t* = 4 y)	0.856 (0.771,0.937)	0.739 (0.561,0.854)	0.760 (0.628,0.911)
AUC (*t* = 5 y)	0.855 (0.771,0.934)	0.780 (0.611,0.905)	0.760 (0.628,0.911)

All values are expressed as accuracy (95% confidence interval) unless otherwise stated

AUC, area under time-dependent receiver-operating curve

**Table 6:** Intraclass coefficient of quantitative image features

Group	Feature	Between-scans	Between operators	Between-algorithms
1	Circularity	0.894	0.797	0.828
	Solidity	0.906	0.754	0.921
2	Variance	0.945	0.965	0.978
	P90	0.989	0.993	0.993
	Auto-correlation	0.971	0.957	0.938
	Sum-average	0.973	0.955	0.933
	Long-run emphasis mean	0.852	0.887	0.887
3	Kurtosis	0.964	0.974	0.990
	Mean	0.980	0.989	0.991
4	Energy	0.980	0.977	0.977
	A_skewness	0.972	0.988	0.993
5	Cluster-shade	0.857	0.696	0.574
6	Maximum-probability	0.962	0.958	0.951
	GLCM Energy	0.939	0.919	0.919
	GLCM Entropy	0.932	0.888	0.888
	GLCM sumEntropy	0.930	0.889	0.888
7	Long-run high gray-level emphasis mean	0.806	0.934	0.950
	Long-run high gray-level emphasis standard error	0.839	0.933	0.952
8	A_Long-run emphasis mean	0.852	0.827	0.907

GLCM, Gray-level co-occurrence matrix

## Abbreviations

ALK: Anaplastic lymphoma kinase
AUCt: Time-dependent area under the receiver operating curve
CI: Confidence interval
CT: Computed tomography
EGFR: Epidermal growth factor receptor
HR: Hazard ratio
ICC: Intraclass coefficient
IQR: Inter-quartile range
KRAS: Kirsten rat sarcoma viral oncogene homolog
LongHEM: Long-run high gray-level emphasis mean
NSCLC: Non-small cell lung cancer
PET: Positron emission tomography
ROI: Region-of-interest
